# Combination of bone marrow mesenchymal stem cells sheet and platelet rich plasma for posterolateral lumbar fusion

**DOI:** 10.18632/oncotarget.19749

**Published:** 2017-07-31

**Authors:** Zunpeng Liu, Yue Zhu, Rui Ge, Jiajun Zhu, Xiaoning He, Xue Yuan, Xinchun Liu

**Affiliations:** ^1^ Department of Orthopedics, First Affiliated Hospital, China Medical University, Shenyang, China; ^2^ Department of Orthopedics, Fourth Affiliated Hospital, China Medical University, Shenyang, China; ^3^ Department of Orthopedics, First Affiliated Hospital, Dalian Medical University, Dalian, China; ^4^ Department of Stomatology, Fourth Affiliated Hospital, China Medical University, Shenyang, China; ^5^ Division of Plastic and Reconstructive Surgery, Department of Surgery, Stanford School of Medicine, Stanford, CA, USA

**Keywords:** BMSC, PRP, rabbit posterolateral spinal fusion, osteogenic ability, fusion rate

## Abstract

Bone tissue engineering provides a substitute for bone transplantation in spinal fusion. This study examined if combined bone marrow-derived mesenchymal stem cells (BMSCs) sheet with platelet-rich plasma (PRP) could promote bone regeneration in a rabbit posterolateral spinal fusion model. BMSCs was isolated and confirmed by Flow cytometric analysis and immunofluorescence staining. The morphology of BMSCs was examined by Hematoxylin and Eosin staining, scanning and transmission electron microscopy. BMSCs were cultured in induction medium or control medium. The osteogenic ability of BMSCs was investigated by various histochemical staining, immunofluorescence staining and qRT-PCR analysis. The BMSCs/PRP was constructed by encapsulating the PRP block with BMSCs sheet. Twenty-four adult rabbits were randomly divided into four groups based on the implanted biomaterials: BMSCs/PRP, BMSCs, iliac crest autograft, and control group. Manual palpation and digital radiography analysis showed that the fusion rate was 100%, 0, 83.3%, and 0 in these 4 groups, respectively. Formation of continuous bone masses in BMSCs/PRP group was confirmed by computed tomography scanning and 3D-reconstruction. These studies demonstrated that BSMCs/PRP significantly accelerated bone regeneration in the rabbit posterolateral spinal fusion model.

## INTRODUCTION

Spinal fusion is one of the most frequently employed surgical techniques for the treatment of various spinal problems including degenerative disc disease with instability, trauma, and deformity. Illiac crest autograft and allogeneic bone are commonly used for lumbar fusion for many years [1]. Following fusion surgery, the most frequent complications are pseudo-arthrosis and donor site diseases [2, 3]. The occurrence of pseudo-arthrosis is high in iliac crest autograft, and about 50% of patients reported the donor site problems such as infection, paresthesis, pain, and hematoma using grid instrumentation [4–6]. Therefore, it is important to find suitable substitutes for spinal fusion surgery. Recently, bone tissue engineering has been extensively studied, and three elements are essential for the bone tissue engineering: 1) the osteo conductive scaffold which facilitates neovascularization and supports bone growth; 2) an osteogenic material which provides cells to add in the newly forming bone; and 3) osteo inductive factors which is capable of inducing osteoblastic differentiation from osteoprogenitor stem cells. Bone marrow-derived mesenchymal stem cells (BMSCs) have been shown to be a promising source for tissue regeneration engineering, as they are easily obtained and can be expanded while maintaining their multi lineage differentiation potential [7]. In the clinical trials, BMSCs have been applied for the treatment of various diseases including graft-versus-host disease, osteogenesis imperfecta and myocardial infarction [8]. In this regard, BMSCs have shown great potential in the application of bone tissue engineering.

Recently, cell sheet technology has been used in tissue engineering to regenerate damaged soft tissues, including corneal epithelia [9], bladder myocardial cells [10], periodontal ligament cells [11], hepatocytes [12] and so on. Cell sheet technology consists primarily of a “thermo-responsive culture dish” which enables reversible cell adhesion to and detachment from the dish surface by controlling the hydrophobicity of the surface [13]. This allows a non-invasive harvest of high-viability cells in an intact monolayer that includes any deposited extracellular matrix, which is important for the structural and adhesive properties. Similarly, BMSCs sheets can be easily generated and transplanted to the site of large bone defects and may serve as a good candidate for bone tissue regeneration.

Platelet-rich plasma (PRP) is an above-baseline concentration of platelets in plasma derived from centrifugation of autologous blood [14]. Platelets in the circulating plasma are found to contain growth factors that function to enhance soft tissues healing. These growth factors are also important for cell proliferation, differentiation, and neovascularization [15]. PRP has also been shown to contain cell adhesion molecules and chemotactic properties that attract fibroblasts and mesenchymal stem cells to the repair site [16]. Therefore, the healing potential of platelets from PRP may be employed to facilitate bone regeneration.

BMSCs combined with various materials such as hydroxyapatite and β-tricalcium phosphate, have been found in the application of bone regeneration using cell suspension system [17, 18]. However, because of the low surface-to-volume ratio of scaffolds, the adhesion rate of BMSCs is very low, which is a big dis-advantage for the cell suspension system. If transplantation of BMSCs without scaffold could enhance bone regeneration at the fusion site, scaffold-free cell-based treatments could be employed, which may represent a better approach to spinal fusion. In this study, we hypothesized that incorporation of an induced BMSC sheet along with PRP would accelerate bone regeneration. We investigated the differentiation of induced BMSCs *in vitro* and the ability of PRP combined with an induced BMSCs sheet in promoting bone regeneration in a rabbit posterolateral spinal fusion model.

## RESULTS

### Isolation of BMSCs

Freshly isolated bone marrow composed of at least three types of cells: erythrocytes, cells of hemopoietic lineage and mesenchymal cells. In the culture, the erythrocytes never adhere to the substrate, and those hemopoietic cells gradually degenerate or do not survive subculturing in this culture condition. The medium changes removed most of the nonadherent cells during the first four days. After 9-10 days of culture, the predominant cells are morphologically homogeneous spindle-shape cells, most of them are mesenchymal stem cells (Supplementary Figure 1).

### Identification of BMSCs with stem cell markers

**Figure 1:**
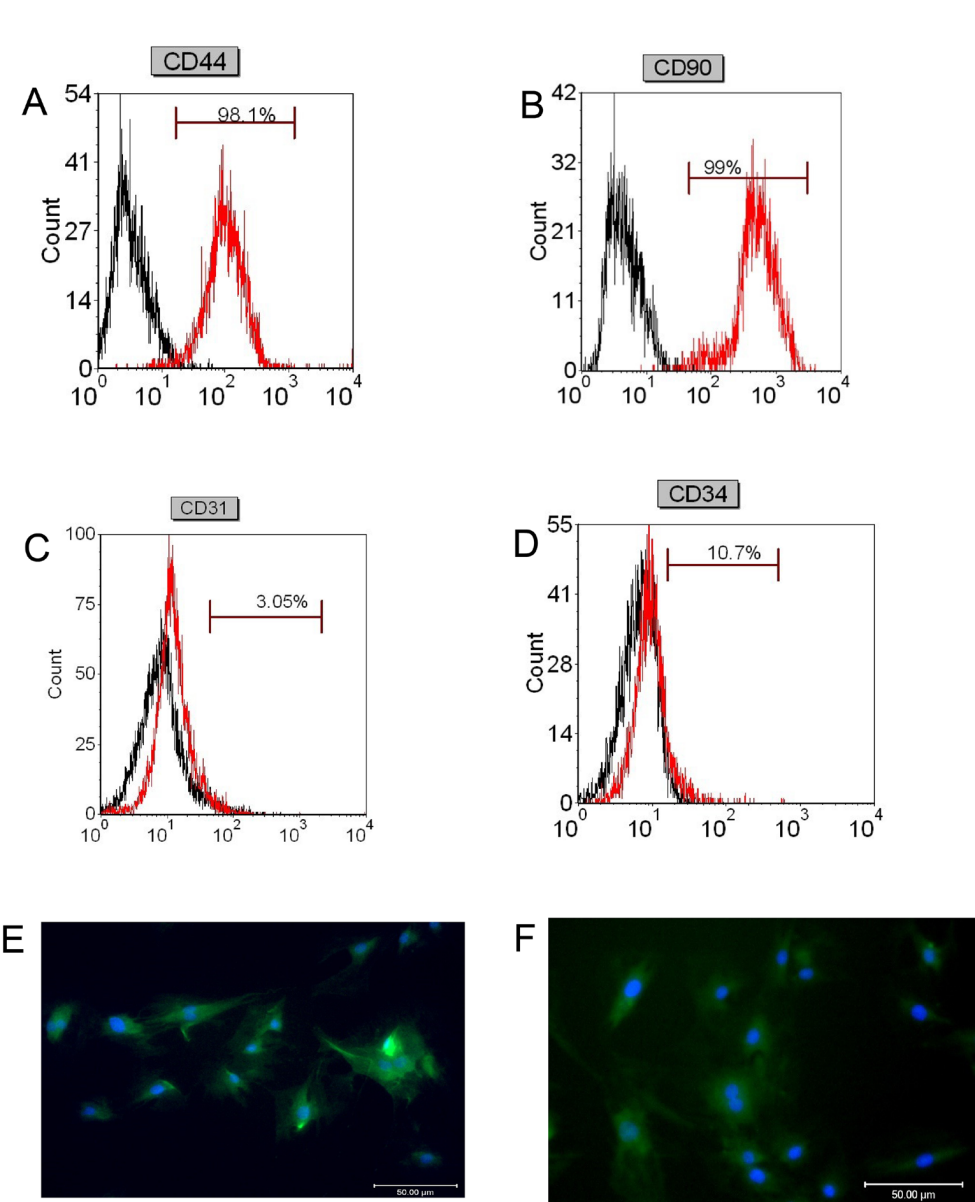
Flow cytometry and immunofluorescence analysis of stem cells markers in isolated BMSCs Flow cytometry analysis of **(A)** CD44, **(B)** CD90, **(C)** CD31, and **(D)** CD34 in isolated BMSCs. Immunofluorescent staining of **(E)** CD44 and **(F)** CD90 in isolated BMSCs. Scale bar = 50μm.

To verify that the isolated cells are truly mesenchymal stem cells, we performed flow cytometry to examine the cell surface marks i.e. CD44, CD90, CD31, and CD34 in the isolated cells. As shown in Figure 1A and 1B, the percentage of CD44 positive cells and CD90 positive cells was 98.1% and 99%, respectively; while the percentage of CD31 positive cells and CD34 positive cells were 3.05% and 10.7%, respectively (Figure 1C and 1D). Immunofluorescent staining confirmed that most of the isolated cells are CD44 positive and CD90 positive (Figure 1E and 1F), indicating that the isolated cells are BMSCs.

### Cell viability and cell growth analysis of BMSCs in normal medium and induction medium

**Figure 2:**
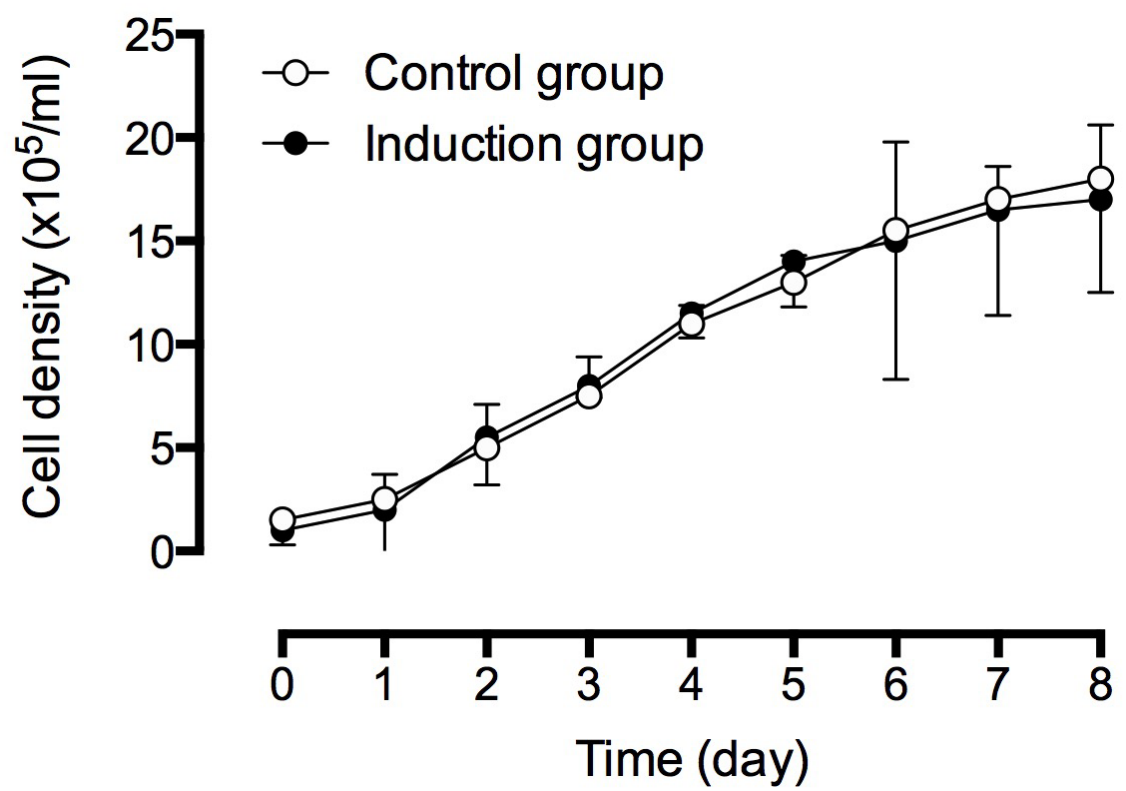
Cell density analysis of BMSCs in control and induced medium The cell density of cells were continuously monitored for eight days. The experiments were performed in three replicates, and data were presented as mean ± SD.

We analyzed the cell viability and cell growth of BMSCs cultured in normal medium and induction medium. The Trypan blue assay showed that there is no significant difference in cell viability between control BMSCs (97.0%) and induced BMSCs (98.0%). The cell growth of BMSCs was determined by measuring the cell density, and as shown in Figure 2, there was no significant difference in the cell density of BMSCs grew in control or induction medium.

### Morphology analysis of BMSCs cultured in normal or induction medium

**Figure 3:**
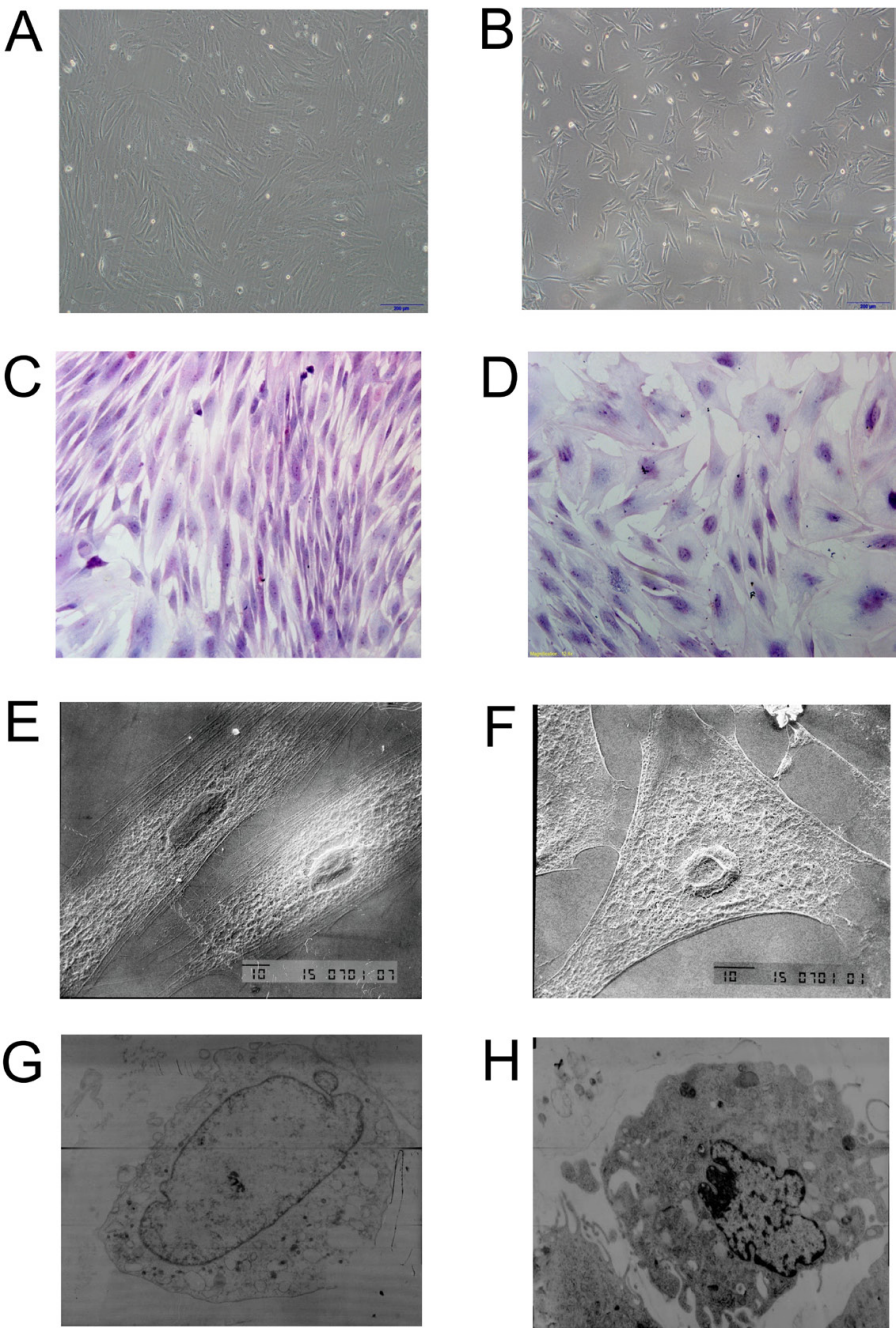
Morphology examination of control BMSCs and induced BMSCs Inverted microscopy of **(A)** control BMSCs and **(B)** induced BMSCs, scale bar = 200 μm. Hematoxylin and eosin staining of **(C)** control BMSCs and **(D)** induced BMSCs, scale bar = 100 μm. SEM images of **(E)** control BMSCs and **(F)** induced BMSCs, scale bar = 10 μm. TEM images of **(G)** control BMSCs and **(H)** induced BMSCs.

BMSCs grown in the control medium were mostly spindle shape with a few polygonal cells. Most of the BMSCs grown in the induced medium were in triangle or polygonal shape (Figure 3A and 3B). Hematoxylin and eosin staining showed that the control BMSCs had basophilic nucleus with clear oval shape, and eosinophilic cytoplasm (Figure 3C); while the induced BMSCs had oval or round nucleus with less eosinophilic cytoplasm (Figure 3D). SEM results showed that the control BMSCs had good cellular morphology with smooth cell surface and spindle shape (Figure 3E); while the induced BMSCs were in triangle or polygonal shapes with clear nucleus, abundant cytoplasm, and the surface of BMSCs showed irregular protrusions, that connect to other cells (Figure 3F). The TEM results showed that the control BMSCs had big nucleus and less cytoplasm with mature organelles and abundant ribosomes and mitochondria (Figure 3G); while the induced BMSCs had increased secretory cells with notch nucleus, and there were calcium particles in the cytoplasm and mitochondria, and increased number of homogenous secretory vesicles and rough endoplasmic reticulum. In addition, the induced BMSCs also had abundant mitochondria and Golgi complex (Figure 3H).

### Osteogenic ability of induced BMSCs

**Figure 4:**
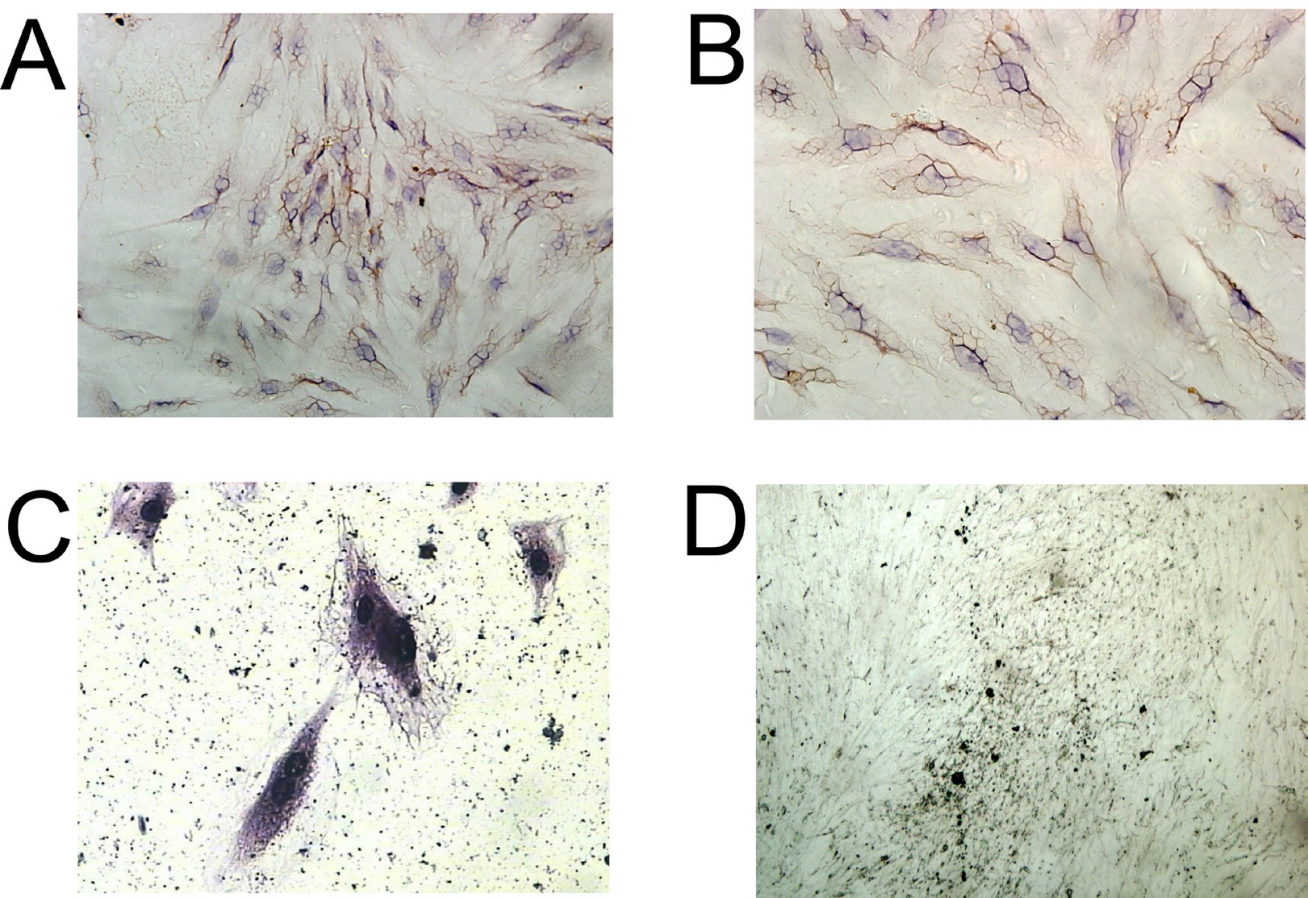
Immunocytochemical analysis of BMSCs Immunocytochemical staining of Collagen I in **(A)** control BMSCs, and **(B)** induced BMSCs, scale bar = 100 μm. **(C)** Gomori staining of induced BMSCs, scale bar = 50 μm. **(D)** Von kossa staining of induced BMSCs, scale bar = 400 μm.

We performed immunohistochemistry to examine the expression of collagen I in the induced BMSCs, and the results showed that most of the cells were collagen I-positive cells after 5 days of culture (Figure 4A and 4B). In addition, Von Kossa staining of induced BMSCs showed large amounts of black calcium nodules in the cell matrix after 14 days of culture (Figure 4D), and Gomori staining of induced BMSCs showed strong ALP expression after 25 days of culture (Figure 4C). These results demonstrated that these cells have the osteogenic ability.

### Morphology and osteogenesis analysis of induced BMSCs sheet

**Figure 5:**
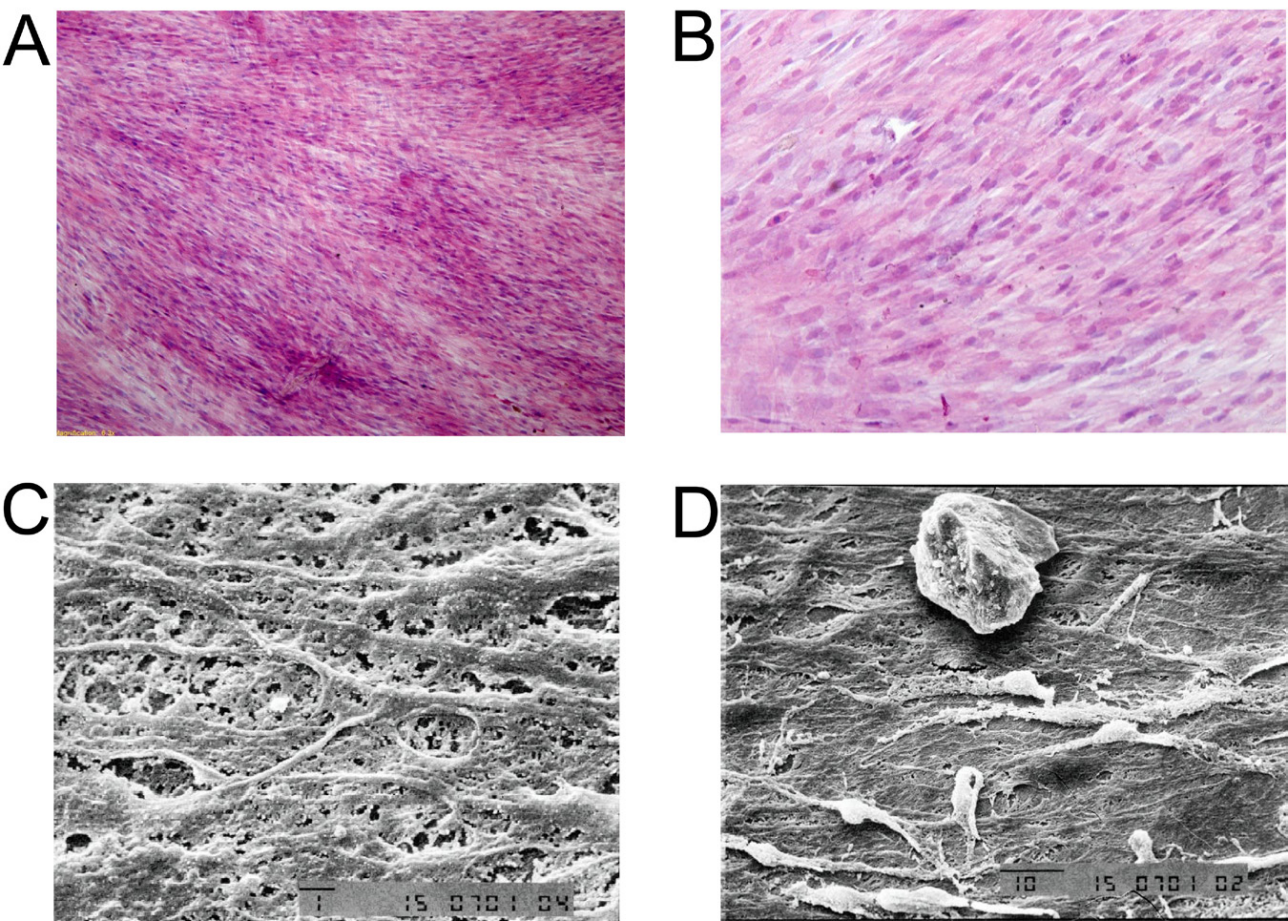
Morphology analysis of BMSCs sheet Hematoxylin and eosin staining of **(A)** BMSCs sheet at 100x, scale bar =400 μm, and **(B)** BMSCs sheet at 200x, scale bar =200 μm. TEM images of **(C)** BMSCs sheet at 1500x, scale bar = 1 μm and **(D)** BMSCs sheet at 1000x, scale bar = 10 μm.

**Figure 6:**
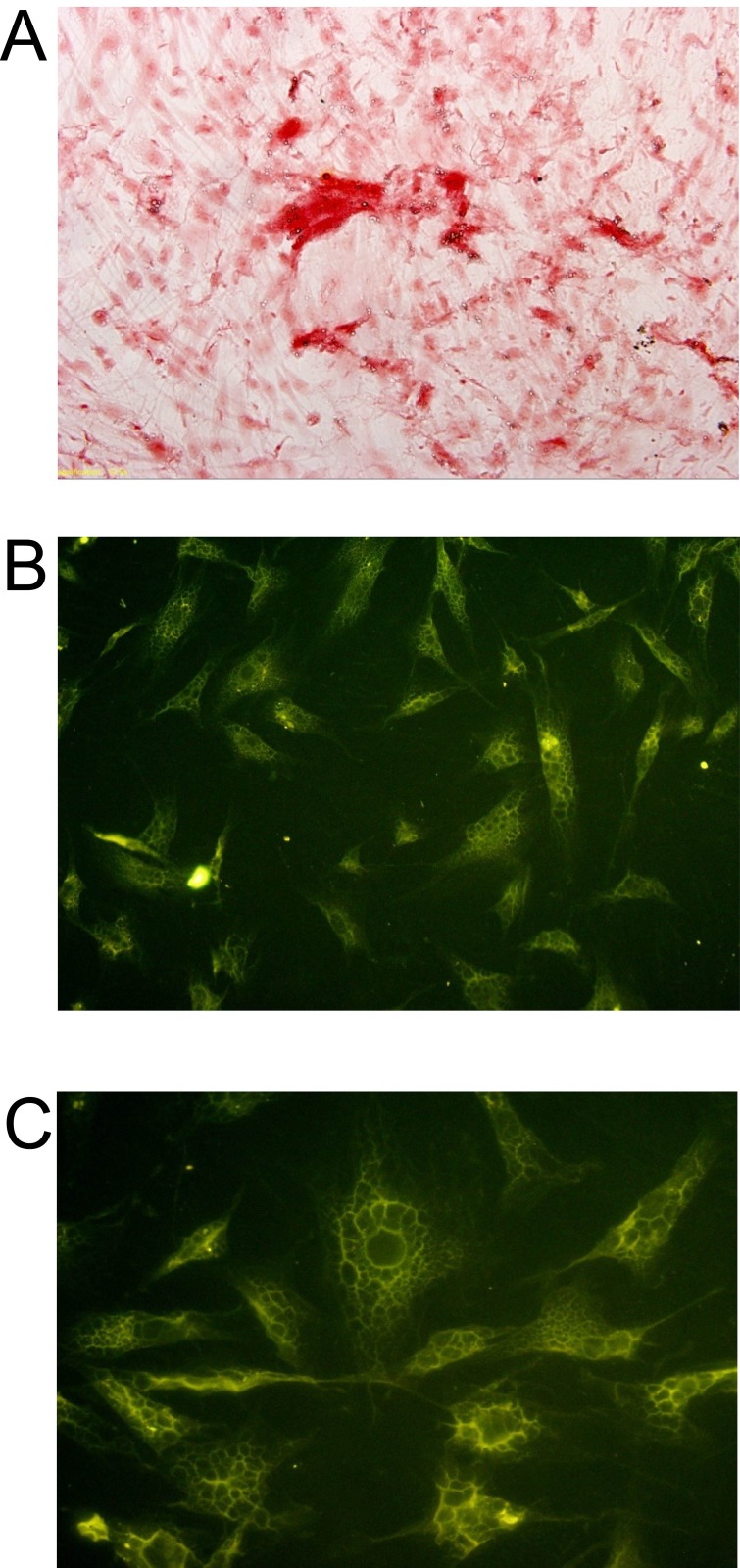
Histochemistry and immunofluorescence analysis of BMSCs sheet **(A)** Alizarin Red staining of BMSCs sheet, scale bar = 200 μm. Immunofluorescent staining of Collagen I **(B)** in BMSCs sheet at 200x, scale bar = 100 μm and **(C)** in BMSCs sheet at 400x, scale bar = 50 μm.

As shown in Supplementary Figure 2, the induced BMSCs formed cell sheets after 3 weeks of culture, and the induced BMSCs sheet can be separated from the petri dish by cell scraper. The hematoxylin and eosin staining showed that the induced BMSCs sheet contained a high density of cells, and the cells were evenly distributed and overlapped between layers (Figure 5A and 5B). The SEM results showed that the induced BMSCs sheet formed by BMSCs and the extracellular matrix with tight connection, and there was calcium salt crystallization (Figure 5C and 5D). As shown in Figure 6A, alizarin red staining showed the large amount of calcium nodules formation in the induced BMSCs sheet. Immunofluorescent staining showed the net-like distribution of collagen I in the induced BMSCs sheet (Figure 6B and 6C). Quantitative RT-PCR results showed the expression of OP, ALP and OC mRNAs in the induced BMSCs sheet (Supplementary Figure 3).

### Manual and radiographic analysis

**Figure 7:**
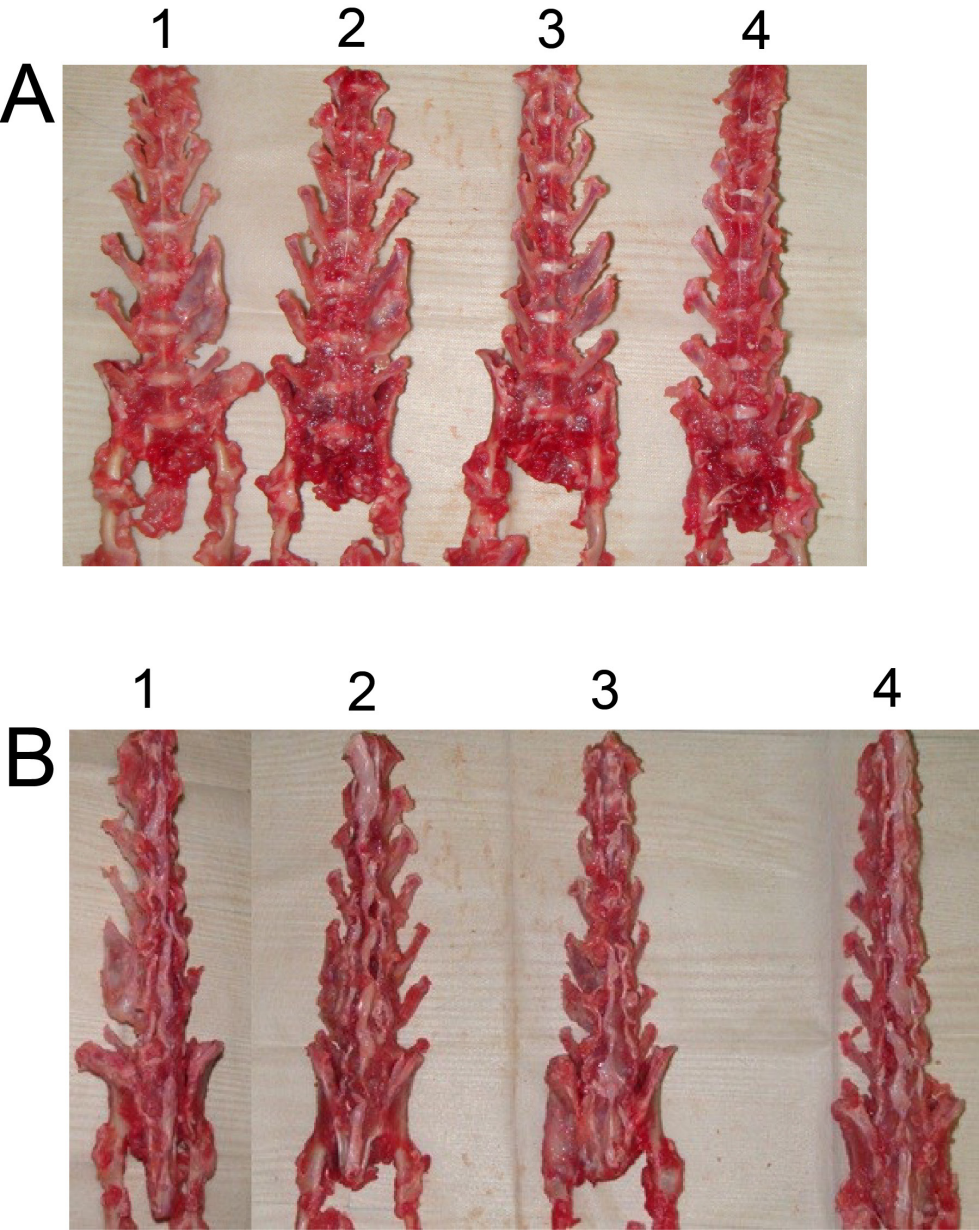
Views of rabbit fusion spines at post-operation 6 weeks **(A)** Anterior view of rabbit fusion spines. **(B)** Posterior view of rabbit fusion spines. 1. BMSCs/PRP group, 2. Iliac crest autograft group, 3. BMSCs group, 4. Control group.

**Figure 8:**
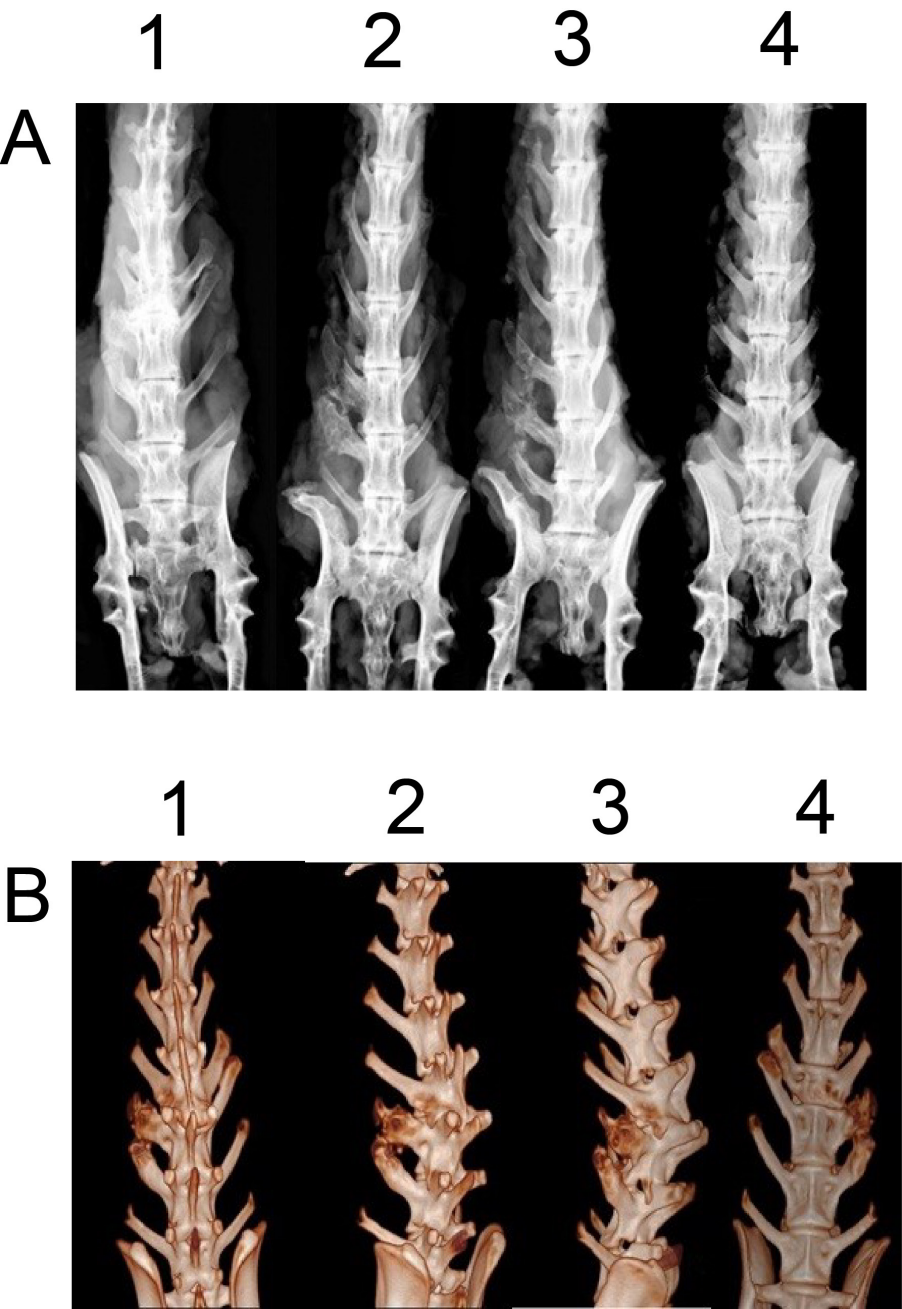
Digital radiography and 3D-CT analysis of rabbit fusion spine at post-operation 6 weeks **(A)** Digital radiographic images of rabbit fusion spines. 1. BMSCs/PRP group, 2. Iliac crest autograft group, 3. BMSCs group, 4. Control group. **(B)** 3D-CT images of lumbar fusion specimens from BMSCs/PRP group.

Six weeks after surgery, excised spines from different groups were assessed for fusion after removal of soft tissue by manual palpation. In both control and BMSCs group, no rabbit had spine fusion (0% fusion) (Table 1). In the iliac autograft group, five out of six rabbits had unilateral spine fusion (83.3% fusion), and in the BMSCs/PRP group, all six rabbits had unilateral spine fusion (100% fusion) (Table 1). Fisher test showed that BMSCs/PRP group had a significant higher fusion rate than both control group and BMSCs group. Gross evaluation of specimens revealed obvious and significant fusion masses in fusion spines from the BMSCs/PRP group and iliac crest autograft groups. The representative images of the rabbit fusion spine from four different groups were shown in Figure 7. Further radiographic analysis of the rabbit fusion spine showed the same fusion rates as examined by manual palpation (Table 1). Representative radiographs of rabbit spines obtained 6 weeks after surgery in each group are shown in Figure 8A.

**Table 1:** Fusion rates (%) assessed with manual palpation and radiographs

Groups	Total (n)	Fusion rate (%) Manual palpation	Fusion rate (%) Radiography
BMSCs/PRP	6	100**^,##^	100**^,##^
BMSCs	6	0	0
Iliac autograft	6	83.3	83.3
Control	6	0	0

Significant differences relative to BMSCs group were indicated as **P<0.01, and significant differences relative to control group were indicated as ^##^*P*<0.01 (Fisher test).

### 3D-CT reconstruction of the rabbit spine

Figure 8B showed that the 3D-CT reconstruction of fusion spine in the BMSCs/PRP group by using 3D-CT. The 3D image showed that the bone mass continuously fused the transverse processes.

### Histological analysis of the spine fusion from BMSCs/PRP group

**Figure 9:**
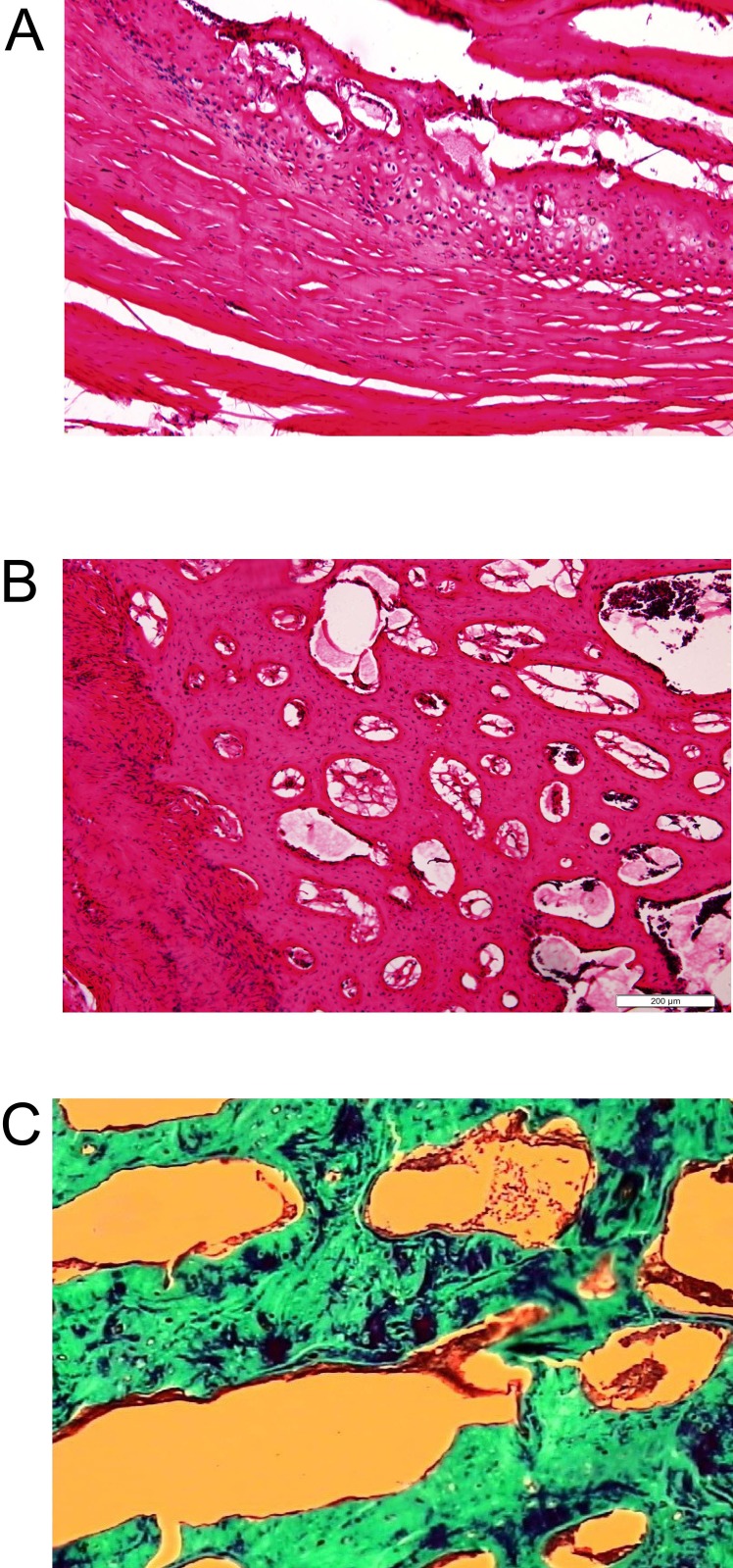
Histology analysis of rabbit spines obtained 6 weeks after surgery Hematoxylin and eosin staining on sections of the L5-L6 transverse processes of the rabbit spine from fusion sample in BMSCs/PRP group at 10x **(A)** and 40x **(B)**. **(C)** Masson’s staining on sections of the L5-L6 processes of the rabbit spines from fusion sample in BMSCs/PRP group.

The histology of the spine fusion in L5-L6 was analyzed by hematoxylin and eosin staining and Masson's staining. The results showed mature bone tissue with bone trabecula and abundant bone matrix in the transverse processes of fusion spines from BMSCs/PRP group (Figure 9A and 9B). Masson's staining demonstrated that abundant collagen was found around the trabecula of the fused spine from BMSCs/PRP group (Figure 9C).

## DISCUSSION

This study showed that incorporation of the induced BMSCs sheet with PRP greatly promoted bone regeneration in the rabbit spinal fusion model. The induced BMSCs sheet and PRP complex can provide cells and growth factors for the bone regeneration. Importantly, our study revealed that BSMCs/PRP complex had a significant positive effect on the bone regeneration in the rabbit spinal fusion model when compared with BMSCs sheet alone.

In practice, the iliac crest autograft or allogeneic bone was commonly used for the bone transplantation to repair bone defect, bone fracture or spinal fusion. Pseudarthrosis and donor site problems are the most frequent complications following fusion surgery, which largely limited its application [2, 4]. Recently, cell-based tissue engineering provided new strategies for providing suitable candidate biomaterials for bone transplantation.

BMSCs are proved to be one of the most promising seed cells for tissue engineering. Studies have found that BMSCs are multi-potential cells that can be induced to differentiate into several mesodermal cell types including osteoblasts, chondrocytes, adipocytes, tenocytes and myoblasts [21]. In the current study, we isolated the BMSCs from the rabbit bone marrow, and flow cytometry and morphology analysis confirmed the success of BMSCs isolation. Furthermore, the osteogenic ability of the isolated BMSCs was induced by culturing the BMSCs in the induction medium, and confirmed by Von Kossa staining, Gomori staining and the immunohistochemical staining of Collagen I. These results were consistent with previous studies showing that the BMSCs have osteogenic potential under suitable induction medium [22]. In previous studies, BMSCs combined with various materials have been found in the application of bone regeneration by using cell suspension system [23, 24]. However, due to the low surface-to-volume ratio of scaffolds, the adhesion rate of BMSCs is very low. Therefore, in the present study, we adopted a cell transplantation method in which BMSCs are cultured and lifted as a cell sheet, which allows a much larger number of cells to facilitate bone regeneration [25]. The BMSCs sheet has been proven effective in bone regeneration and can effectively preserve cell-to-cell contact and the extracellular matrix [26]. In addition, the layered cell sheet may mimic the *in vivo* bone deposition of bone matrix where osteoblasts are attached on the mineralized sheet [16]. In this study, The BMSCs sheet can be easily detached from the petri-dish, and more importantly, the SEM results showed that the extracellular matrix as well as cell-cell interactions remain intact. The immunofluorescence results showed that collagen I was extensively distributed in the BMSCs sheet. In addition, the alizarin red staining was also found to be highly positive. Quantitative RT-PCR analysis further showed the expression of OP, ALP and OC mRNAs in the induced BMSCs sheet. These results suggest the osteogenic differentiation of the induced BMSCs sheet.

PRP, which can be easily obtained from blood, is a natural source of growth factors, including platelet derived growth factor, transforming growth factor, insulin-like growth factor, and vascular endothelial growth factor, that can induce osteogenic differentiation of BMSCs [27]. Previous studies showed that BMSCs from PRP-treated mouse had high level of Collagen I and ALP activity [28]. Though the specific growth factors in the PRP was not identified in this study, previous study has shown that the levels of growth factors in PRP samples from goat were higher than those of circulating plasma and PRP can promote the osteogenic differentiation of BMSCs [29, 30]. Owing to the positive effects of BMSCs sheet and PRP on the osteogenesis, we constructed the BMSCs/PRP biomaterials in which the PRP blocks were wrapped by the induced BMSCs sheet and examined the bone regeneration using a rabbit posterolateral spinal fusion model. Manual palpation and radiographic analysis as well as 3D-CT reconstruction of the fused spine showed that BMSCs/PRP significantly promoted the regenerative response and greatly accelerated bone regeneration compared to other control groups in the rabbit posterolateral spinal fusion model.

One weakness of the present study was that mechanical tests were not performed. In our study, the iliac crest autograft group also had high rate of spinal fusion, and its fusion rate is similar to that in BMSCs/PRP group. However, whether the mechanical property of fused spine in BMSCs/PRP group is similar to or better than that of iliac crest autograft group is unknown. Future study will be performed to examine the mechanical properties of these fused spines.

In summary, our data suggested that the implanted induced BMSCs sheet and PRP play crucial roles in promoting bone regeneration. Our procedure is simple and safe with minimal side effects because both BMSCs sheet and PRP are autologous, nonimmunoreactive and nontoxic. This procedure may be applicable for spinal fusion and could represent a novel alternative to autologous or allogenic bone grafts.

## MATERIALS AND METHODS

### Animals

Adult Japanese white rabbits (3 – 5 kg) were purchased from the Animal Department of China Medical University. All animals were housed and treated in strict accordance with the Guidelines on the Care and Use of Laboratory Animals issued by the Chinese Council on Animal Research and the Guidelines of Animal Care. All animal procedures were approved by the China Medical University Laboratory Animal Care.

### BMSCs isolation and culture

Rabbit BMSCs were obtained from the femurs of anesthetized adult Japanese white rabbits. In brief, 10 ml bone marrow was diluted 1:2 with phosphate buffer solution (PBS) and loaded in a 5 ml Percoll (density, 1.077; PHARMACIA). Cells were harvested from the interface after centrifugation at 2,000 rpm for 20 min and washed with Dulbecco’s modified Eagle’s medium (DMEM, Gibco, Grand Island, NY). Cells were re-suspended in Low glucose DMEM containing 10% fetal bovine serum (HYCLONE), 100 U/ml penicillin, 100 g/ml streptomycin, and incubated at 37°C. When a monolayer was formed in the primary culture, cells were sub-cultured for further experiment.

### Induction medium preparation

The induction medium was prepared as follow: High glucose DMEM, 10% fetal bovine serum (FBS), 50 mg/L Vitamin C, 10 mM β-sodium glycerophosphate, 100 nM dexamethasone, 100 U/ml penicillin, 100 g/ml streptomycin. The induction of BMSCs was achieved by sub-culturing the isolated BMSCs in the induction medium.

### Preparation of BMSCs sheet

BMSCs in the third passage was seeded onto the 10 cm petri-dish with a density of 1x10^6^/cm^2^, and the BMSCs were cultured with Low glucose DMEM supplemented with 10% FBS for 2 days, then the BMSCs were cultured under the induction medium for 3 weeks without passage, with the induction medium refreshed every 2 days. After 3 weeks of culture, the semi-transparent thin sheet was observed at the bottom of the petri-dish (Supplementary Figure 2B). The BMSCs sheet was separated from the petri-dish by a cell scraper (Supplementary Figure 2C).

### Flow cytometric analysis of BMSCs using CD44, CD90, CD31 and CD34 biomarkers

For the flow cytometric analysis, fluorescein isothiocyanate (FITC) conjugated antibodies against CD44, CD90, CD31, and CD34 (Dako corporation, Glostrup, Denmark) were used. Isolated cells cultured in the induction medium from the third passage were used. First, cells attached to the bottom of the flask were detached by Trypsin/EDTA treatment. Then, the appropriate concentration of the above-mentioned antibodies (1:10 dilution) was added, and incubated at room temperature for 20 min at dark. After that, the cells were washed with PBS and analyzed by flow cytometry using the FITC method and a FACS Calibur (Becton Dickinson).

### Immunofluorescent staining for BMSCs cells

The BMSCs cells were treated with 0.3% Triton, and were blocked at room temperature for 90 min before incubation with FITC-conjugated antibodies against CD44 and CD90 at 4°C for 30 min. Then the cells were stained with 4',6-diamidino-2-phenylindole at room temperature for 10 min. The fluorescent signals were examined under a fluorescent microscope.

### Cell viability and cell growth assays

Isolated cells from the third passage were used for the assays. Cell viability was examined using the tryphan blue assay. 1×10^5^/ml cells were seeded on the six-well plate and cultured with either normal medium or induction medium. After culture for three days, 20μl of cell suspension was mixed with 80 μl lysing solution (Beckman Coutler, Marseille Cedex, France) for 5 min, and then mixed with 100 μl tryphan blue solution (0.4% w/v, GIBCO). The samples were loaded into a Neubauer hemocytometer, and the cells in each 1 mm^2^ area were counted under a light microscope. For the cell growth assay, 1×10^5^/ml cells were seeded on the 48-well plate and were cultured with either Low glucose DMEM medium or induction medium. The cell number was counted under a light microscope for consecutive 8 days.

### Hematoxylin and eosin staining

The Hematoxylin and eosin staining of isolated BMSCs and BMSCs sheets were performed according to previous protocol [19].

### Scanning electron microscopy (SEM)

The specimens were submerged in fixative (2.5 % glutaraldehyde in phosphate buffer at pH = 7.3) overnight. Specimens were dehydrated using graded alcohol and sputter coated with gold. SEM imaging of specimens was conducted at various magnifications.

### Transmission electron microscopy (TEM)

The specimens were fixed in 2.5% glutaraldehyde in 0.1M phosphate buffer for 2 hours. The samples were washed with 0.1M phosphate buffer, post-fixed in 1% OsO4 buffered with 0.1M phosphate buffer for 2 hours, dehydrated in a graded series of ethanol and embedded in Epon 812. Specimens were collected on copper grids, double-stained with uranyl acetate and lead citrate, and then examined by transmission electron microscopy.

### Immunocytochemical staining and immunofluorescent staining of collagen I

The immnuocytochemical staining was performed to examine the distribution of collagen I in the induced BMSCs. Briefly, the BMSCs were fixed in formalin for 20 min and incubated with 1% H_2_O_2_ for 10 min. After that, the samples were blocked with goat serum for 30 min at room temperature, followed by incubation with primary antibody (anti-collagen I mouse monoclonal antibody) at 4°C overnight. After primary antibody incubation, cells were further incubated with the HRP-conjugated secondary antibody, and the immnuocytochemical staining was visualized by incubation with 3,3'-Diaminobenzidine. Images of the immunocytochemical staining were examined under a light microscope.

For immunofluorescence staining, BMSCs sheet were fixed with 4% paraformaldehyde for 10 min, and blocked for 30 min in 5% normal goat serum. After that, samples were incubated with primary antibody(anti-collagen I mouse monoclonal antibody) for 12 hours. Then, they were incubated with secondary antibody (FITC conjugated secondary antibody, Sigma, F0382) for 2 hours and the nucleus was subsequently counterstained by DAPI. The slides were mounted with mounting medium, and the fluorescent signals were examined under a fluorescent microscope.

### von Kossa staining

The presence of mineralized deposits in induced BMSCs cultures was demonstrated with von Kossa staining. The induced BMSCs were rinsed twice with Tyrode's salt solution, fixed with 1% glutaraldehyde (v /v) for 15 min, and rinsed three times with distilled water. One ml of 2% (w/v) silver nitrate (Sigma Chemical) was added per dish following 10 min incubation in a dark environment. Cultures were then rinsed 3X times with distilled water and exposed to bright light for 15 min. Cultures were rinsed and then dehydrated with 100% ethanol.

### Gomori staining

Induced BMSCs were fixed in cold 10% neutral buffer formalin for 1 hour at 4°C. Then, the cell layers were washed with deionized water and allowed to air dry. The fixed cells were incubated with buffer containing 0.1 mg/ml naphthol AS-MX phosphate disodium salt (Sigma) and 0.6 mg/ml Fast Red TR salt (Sigma). After 1 hour at 37°C, the cell layers were washed with deionized water and observed both grossly and with the light microscope.

### Alizarin red histochemical staining

The BMSCs sheet was fixed in cold 70% ethanol for 1 hour at 4°C. Then, the cell layers were washed with deionized water and allowed to air dry. The fixed cells were stained with 2% Alizarin red pH 7.2 (Sigma). After 1 hour at 37°C, the cell layers were washed with deionized water and observed both grossly and with the light microscope.

### Quantitative RT-PCR

Total RNA was extracted from the cells using TirZol reagent. The extracted RNA was then reverse-transcribed into single stranded cDNA, using MMLV Reverse Transcriptase and Oligo(dT) primers according to the manufacturer’s instructions (Promega). For gene expression analysis, real-time PCR was performed using SYBR-Green-based protocols in an ABI Step One system. The expression levels of target genes in each sample were calculated after being normalized to the Ct value of the beta-actin housekeeping gene. The primers for respective genes include osteopontin(OP), alkaline phosphatase (ALP) and osteocalcin (OC)were shown in Table 2.

**Table 2:** Primers for qRT-PCR

Genes	Primer sequence
*Beta-actin*	5’-ATCGTGCGGGACATCAAGGA-3’(F)
	5’-CAGGAAGGAGGGCTGGAACA-3’(R)
*Osteocalcin*	5’-TCTACCAGTTGCAGCCTGAC-3’(F)
	5’-GTTCCCTTCCTCCTTGATTT-3’(R)
*Osteopontin*	5’-ACAATATAAGCGCGAGGCCA-3’ (F)
	5’-GCTCGATGGCTAGCTTGTCT-3’ (R)
*ALP*	5’-GAGGATGAGAGCAAGGACCC-3’ (F)
	5’-CCGCAGTCGGTGTAGGG-3’(R)

### Preparation of PRP and implanted biomaterials

Autologous PRP was prepared as described previously [20]. Briefly, 10 mL of whole blood was drawn from the marginal auricular vein using an 18-gauge catheter. The blood was injected into a sterile centrifuge tube containing 1.5 mL of sodium citrate. The mixture was centrifuged at 1200×g for 10 min to separate the plasma from the red blood cells. The plasma was centrifuged again at 2500×g at 4°C for 20 min, and the precipitated platelets (1 mL) were collected. The prepared autologous PRP was settled in a customized mold (Supplementary Figure 4A), and the PRP was placed on the BMSCs sheet (Supplementary Figure 4B). Then the implanted biomaterials were prepared by wrapping the PRP with the detached BMSCs sheet.

### Surgical procedures for biomaterial implantation

Rabbits (n = 24) were subcutaneously injected with buprenoprhine 30 min before surgery, and the surgical site was shaved and prepped with betadine and 70% ethanol. The rabbits were anesthetized with intravenous injection of 25 mg/kg sodium pentobarbital. Aseptic technique was used for all surgical procedures. The L6 vertebral body was identified using the iliac crest as a landmark. A 6-cm longitudinal midline incision was made through the skin and subcutaneous tissue over L5–L6 down to the lumbodorsal fascia. A 3-cm longitudinal paramedial incision was then made in the paraspinal muscles bilaterally to expose the transverse processes of L5 and L6, which were decorticated with a high-speed burr. The surgical site was then irrigated with sterile saline, and 2 cm X 1.5 cm X 0.5 cm pieces of biomaterial (BMSCs/PRP group, iliac crest autograft group, BMSCs group, control group) were placed unilaterally (Supplementary Figure 4C and 4D), with each implant spanning the transverse processes. All the implanted biomaterials were randomly assigned to each animal. The implants were then covered with the overlying paraspinal muscles, and the lumbodorsal fascia and skin were closed with 4–0 Prolene sutures (Ethicon, Inc., Somerville, NJ, USA). Animals were allowed to ambulate, eat, and drink ad libitum immediately after surgery.

### Manual assessment of fusion

Six weeks post surgery, animals were euthanized and the spines were surgically removed and blindly evaluated by three independent observers for motion between levels after removal of soft tissue. Nonunion was recorded if motion was observed between the facets or transverse processes on surgical side. Complete fusion was recorded if no motion was observed. Spines were scored as either fused or not fused. Unanimous agreement was required to consider a spine completely fused.

### Radiographic assessment of fusion

Radiographs of the lumbar spine were taken for each animal at 6 weeks after surgery using a Faxitron LX60 cabinet radiography system for posteroanterior images. Radiographs were blindly evaluated by three independent observers. Spines were scored as either fused or not fused. Unanimous agreement was required to consider a spine completely fused.

### Computed tomography (CT) and three-dimensional (3D) reconstruction

The spine from BMSCs/PRP group was scanned by CT using LightSpeed 16 (GE, Milwaukee, WI), and the data were collected at 80 kV and 300 mA, and reconstructed using the cone-beam algorithm.

### Histological analysis

Six weeks post implantation, the spines were dissected, and the specimens were fixed in 40% ethanol, decalcified using standard 10% decalcifying solution HCl (Cal-Ex; Fischer Scientific, Fairlawn, NJ), washed with running tap water, and then transferred to 75% ethanol. Serial coronal sections between the L5-L6 transverse processes were cut carefully at the left sides. The specimens were embedded in wax for sectioning. Coronal sections (5 μm) were cut from the paraffin blocks using a microtome (LS-113; DAIWA-KOKI, Saitama, Japan). The sections were then stained with hematoxylin and eosin for basic morphology or Masson (Masson kit, Zhuchun, Co.Ltd., Shanghai) for collagen morphology.

### Statistical analysis

The statistical analyses were performed using the SPSS software package (Version 12.0). The differences among groups were analyzed by Chi-square test. All data are expressed as mean ± SD. Differences were considered significant when P<0.05.

## SUPPLEMENTARY MATERIALS FIGURES


