# Supplementary material for: Combination of bone marrow mesenchymal stem cells sheet and platelet rich plasma for posterolateral lumbar fusion
Source: Oncotarget. 2017 Jul 31;8(37):62298–311. doi: 10.18632/oncotarget.19749 (PMC5617506; doi:10.18632/oncotarget.19749)
Supplement: Supplementary file 1 [file oncotarget-08-62298-s001.pdf]

Combination of bone marrow mesenchymal stem cells sheet and platelet rich plasma for posterolateral lumbar fusion

SUPPLEMENTARY MATERIALS

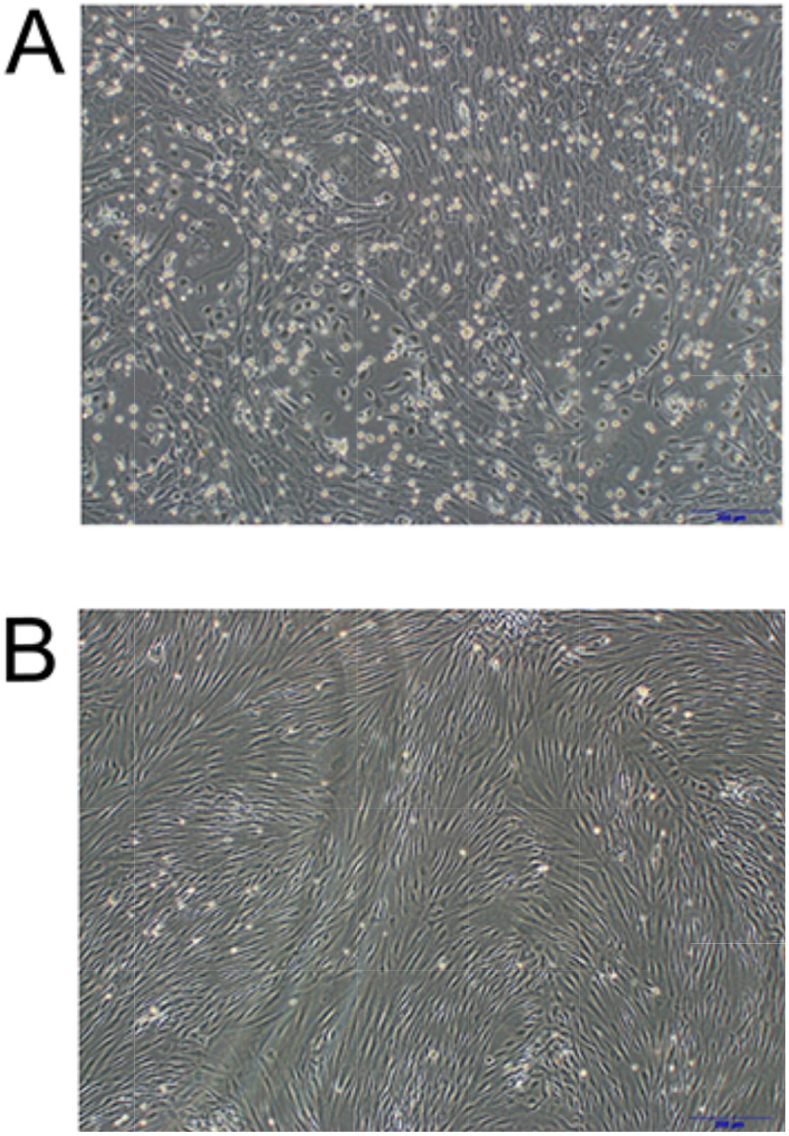

Supplementary Figure 1: Morphology of isolated BMSCs cultured for 4 days (A) and 10 days (B).

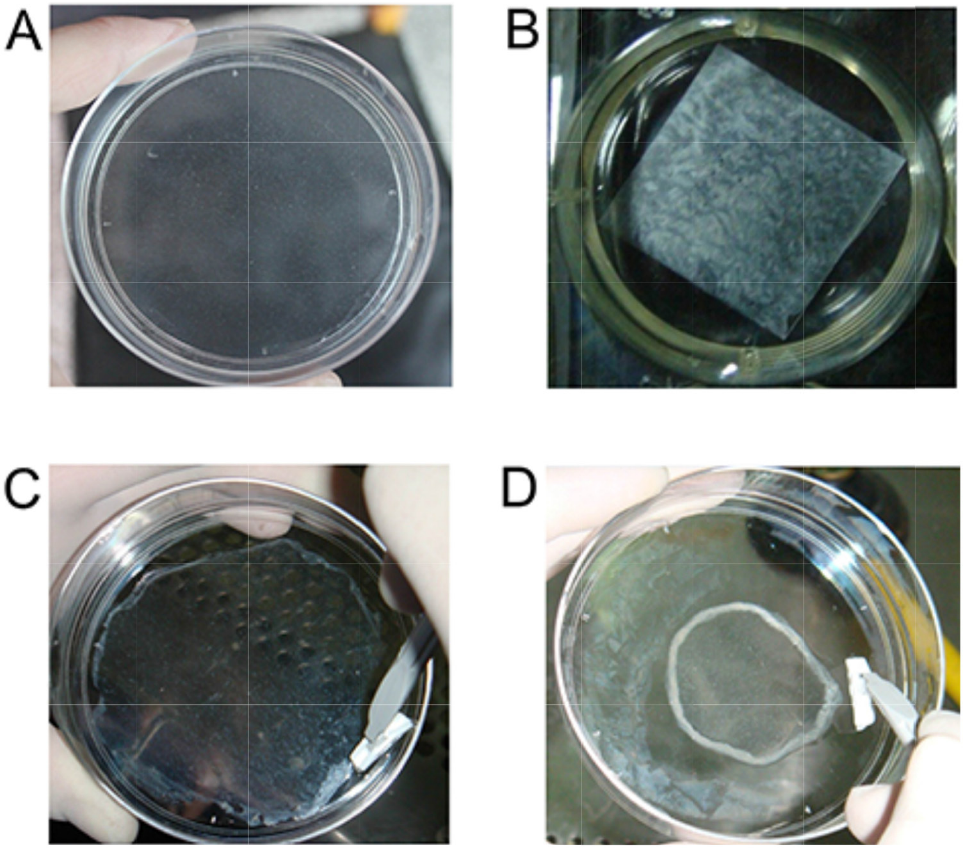

Supplementary Figure 2: Preparation of BMSCs sheet.

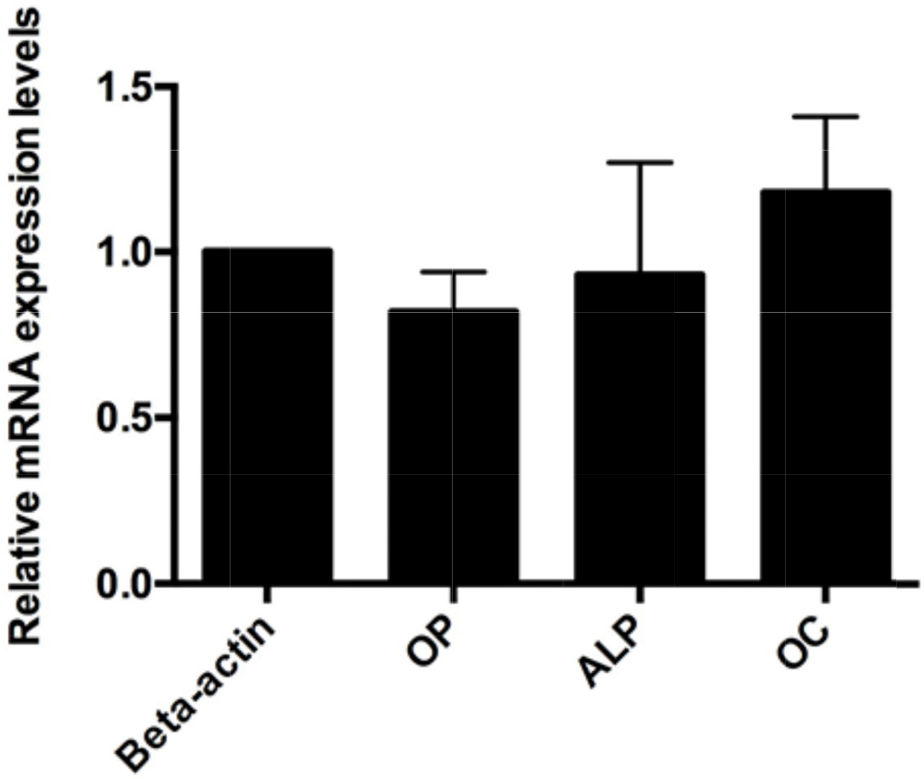

Supplementary Figure 3: Relative mRNA levels of OP, ALP and OC as normalized to beta-actin.

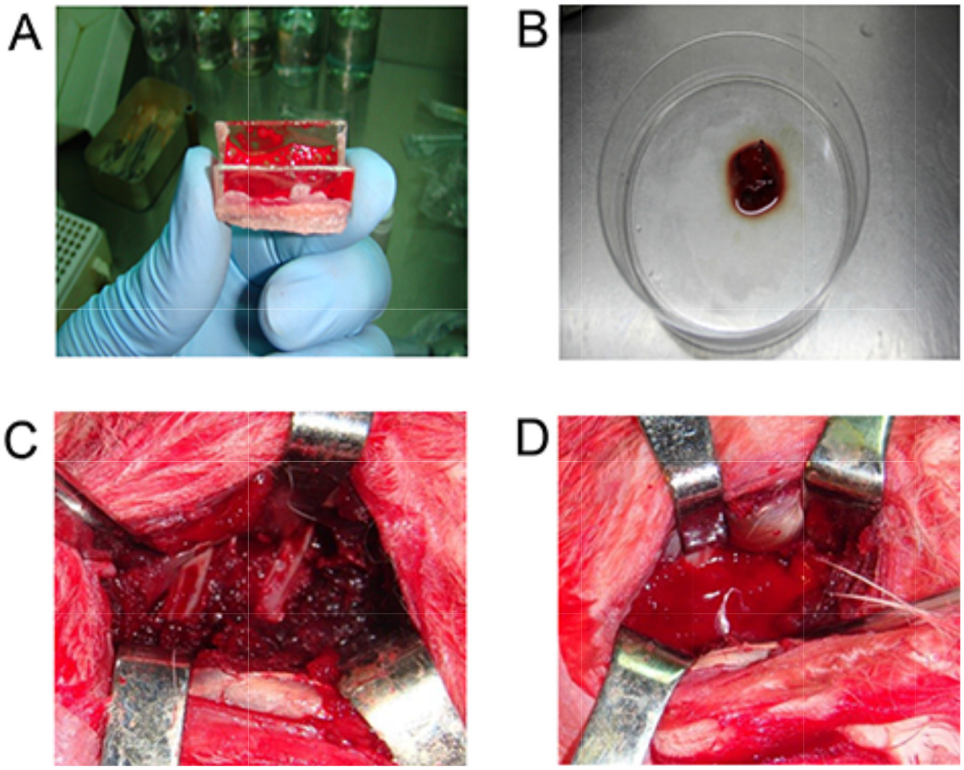

Supplementary Figure 4: Preparation of PRP and implanted biomaterials.
